# Calcium Alginate Aerogel-MIL160 Nanocomposites for CO_2_ Removal

**DOI:** 10.1021/acs.langmuir.5c00143

**Published:** 2025-05-08

**Authors:** Hamed Yousefzadeh, Aysu Yurdusen, Ayça Tüter, Gokhan O. Aksu, Georges Mouchaham, Seda Keskin, Christian Serre, Can Erkey

**Affiliations:** † Department of Chemical Engineering, 52998 Yeditepe University, Atasehir, Istanbul 34755, Türkiye; ‡ Department of Chemical and Biological Engineering, 52979 Koç University, Rumelifeneri Yolu, Sariyer, Istanbul 34450, Türkiye; § Institut des Matériaux Poreux de Paris, ESPCI Paris, 26909 Ecole Normale Supérieure, CNRS, PSL University, Paris 75005, France; ∥ Koç University TÜPRAŞ Energy Center (KUTEM), Koç University, Rumelifeneri Yolu, Sariyer, Istanbul 34450, Türkiye; ⊥ Koç University Hydrogen Technologies Center (KUHyTech), Koç University, Rumelifeneri Yolu, Sariyer, Istanbul 34450, Türkiye

## Abstract

Using MOFs in powder form leads to mass transfer limitations and large pressure drops in packed bed adsorbers. Use of MOF/aerogel composites (called MOFACs) in bead form could overcome these challenges without compromising the MOF’s adsorption performance, as observed with other shaping methods, such as the use of polymeric binders. In this study, Ca-alginate-aerogel-MIL-160­(Al) MOFACs (AlgMIL160) were prepared via sol/gel-assisted direct mixing methods, followed by supercritical drying. The gas sorption, powder X-ray diffraction, FTIR, and scanning electron microscopy characterization results showed that the MOF was successfully incorporated into the aerogel, while the MOF structure was preserved. Adsorption measurements were carried out in both static single-component and dynamic binary gas mixture modes. Obtained isotherms were successfully fitted to the Langmuir model followed by ideal adsorbed solution theory (IAST). The single-component gas adsorption isotherms of CO_2_ on MOFACs with MIL-160­(Al) loadings of 25, 50, and 75 wt % revealed a CO_2_ uptake of 0.43, 0.70, and 0.98 mmol/g at 150 mbar and 25 °C which were higher than that of pure MOF (1.23 mmol/g) based on the MOF loading in the composites, showing the synergistic effect of aerogel and MOF composites. Incorporation of MIL-160­(Al) into the aerogel network which is comprised of 75% MIL-160­(Al) and 25% Ca-alginate aerogel enhanced MIL-160­(Al)’s CO_2_/N_2_ IAST selectivity from 53 to 70 at 25 °C and 1000 mbar. Both experimental and simulated CO_2_ adsorption isotherms showed good agreement. The dynamic adsorption performance of the MOFACs studied by using a binary mixture of 15% CO_2_/85% N_2_ was close to the single-component CO_2_ adsorption with slightly decreased uptake showing the competitive adsorptions between CO_2_ and N_2_ molecules. This novel nanocomposite with remarkable CO_2_ capture performance can be used in gas adsorbers without causing large pressure drops.

## Graphical abstract

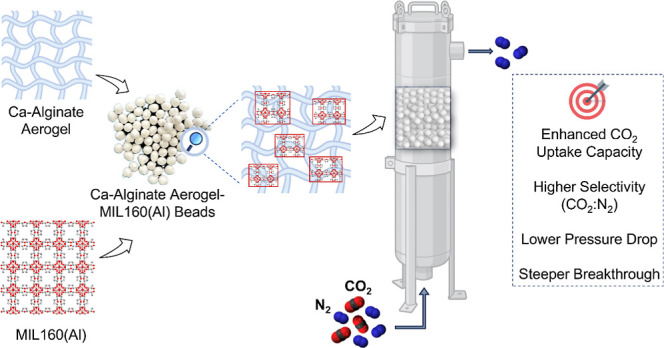

## Introduction

Industries with high carbon footprints, such as cement, steel, and chemical manufacturing, contribute significantly to global CO_2_ emissions. In 2024, these emissions reached an alarming value of 37.8 Gt.^1^ Hence, there is a significant need for emission reduction measures and viable technologies that can effectively capture CO_2_ from flue gas and process streams within these sectors. Conventional capture methods based on gas absorption, predominantly relying on amine-based solvents, encounter challenges related to solvent degradation during the regeneration process and high process costs.^2,3^ Adsorption is an alternative promising technology that is under development. The commercialization of adsorption-based technologies requires the development of effective adsorbent materials with desired properties such as notable CO_2_ uptake and selectivity, reusability over a wide range of pressures and temperatures, high multicycle mechanical stability, and good regeneration properties.^4–7^ Adsorption relies on weak interactions between the adsorbate molecules and the adsorbent surface, facilitating the energy-efficient regeneration, while effective adsorbents offer rapid diffusion, recyclability, long-term stability, and selective CO_2_ adsorption.^8^ Zeolites and activated carbons have been extensively studied for the adsorption of CO_2_ and reported as efficient adsorbents. However, the performance of zeolites and activated carbons is hindered in the presence of moisture and at higher temperatures.^9^

Metal–organic frameworks (MOFs) are crystalline materials that possess a highly ordered three-dimensional framework created through the coordination of metal ions or metal oxide clusters with organic ligands.^10–14^ A range of unique properties such as high porosity, tunable pore structure, high specific surface area, chemical and thermal stability, and surface functionality makes MOFs attractive for various applications such as catalysis,^15^ gas storage and separation, CO_2_ capture,^16–18^ water and wastewater treatment,^19^ sensors,^20^ and drug delivery.^21^ The adsorption capacity and selectivity of a gaseous species over a MOF depend on the strength of the interaction between the adsorbate and adsorption sites as well as the textural properties of the framework.^9^ MOFs, such as Mg-MOF-74,^22,23^ Cu-BTC (HKUST-1),^24,25^ UTSA-16,^26^ MIL-101-(Cr),^27,28^ and MIL-160­(Al),^29^ CALF-20,^30^ MIL-120­(Al),^31^ MUF-16 ^32^ (HKUST stands for Hong Kong University of Science and Technology, UTSA stands for University of Texas at San Antonio, MIL stands for Materials for Institut Lavoisier) have been reported to offer high uptake and selectivity toward CO_2_ in the recent years.

Although MOFs possess intriguing properties and exhibit industrial potential for CO_2_ capture, they can typically be manufactured in the form of a fine powder. This characteristic can decrease efficiency of MOFs in CO_2_ capture, due to a wide variety of factors such as dust formation, high pressure drops, and the risk of entrainment in packed beds.^33^ Therefore, to increase the usage of MOFs in adsorption columns, innovative design of MOF-based materials is needed to integrate nanoscale MOF structures into bulk components with specific geometry to overcome the above-mentioned challenges in industrial applications.^34,35^ MOFs are usually shaped into monoliths, tablets, foams, and granules using mechanical techniques (i.e., pressing, granulation, and extrusion) and wet granulation with chemical binders. However, these methods may damage the crystallinity of MOFs, decrease the surface area, block the pores of MOFs, and thus reduce CO_2_ uptake.^36–42^ HKUST-1, Mg-MOF-74, and MIL-101-Cr, which were considered as promising CO_2_ adsorbents, were pelletized using binders. When PVB binder was used for pelletization, the CO_2_ uptake decreased compared to the original MOF powder. For instance, MIL-101-Cr powder exhibited a CO_2_ uptake of 2.23 mmol/g (mmol of adsorbed CO_2_ per gram of the adsorbent) at 25 °C and 1 atm, whereas the pelletized form with 95% MOF loading showed an uptake of 1.98 mmol/g instead of the expected 2.12 mmol/g.^43^ In another study, Mallick et al. reported that using 10% PMMA (a glassy polymer binder) reduced the CO_2_ adsorption efficiency of NbOFFIVE-1-Ni beads by around 11%, while using 10% PEG (a rubbery polymer binder) caused a much higher reduction of 26%. Although it was observed that using glassy polymers does not significantly alter the CO_2_ adsorption behavior with respect to MOF loading, a reduction in CO_2_ uptake was noted for rubbery polymers.^44^ Therefore, there is still a demand for pioneering techniques for shaping MOFs where the properties of MOFs are not adversely affected.

Incorporating MOFs into meso/macro-porous aerogel matrices to make MOF/aerogel composites (MOFACs) is an attractive strategy to achieve highly desirable products.^45^ Organic or inorganic aerogels with a 3D nanoporous network structure exhibit unique properties including low density, high surface area, large pore volume, high tunability, high porosity, and low thermal conductivity enabling excellent performance in gas adsorption, catalysis, water treatment, and thermal insulation.^46–49^ Aerogels can be synthesized from different precursors in various forms such as monoliths, thin sheets, and beads depending on the application.^50^ The combination of the ordered microporosities of MOFs suitable for CO_2_ capture with the meso- and macro-porosities of aerogels makes MOFACs hierarchically multimodal porous materials with morphological, mechanical, physicochemical, and functional properties superior to the aerogel and the MOF. The synthesis of MOFACs typically involves incorporating MOFs into the aerogel matrix where the MOF particles are the dispersed phase, while the aerogel matrix serves as the continuous phase.^51^ Unlike polymer binders, where the performance is adversely affected, the incorporation of MOFs into aerogels not only maintains the adsorption performance of MOFs but also enhances it through synergistic effects, resulting in improved adsorption capabilities. Recently, Zhao et al.^52^ demonstrated a novel strategy to develop hierarchical structure of HKUST-1 aerogel where crystalline particles of HKUST-1 were grown on Armid nanofibers of (ANFs) aerogel. This MOFAC achieved a CO_2_ adsorption capacity of 7.29 mmol/g at 25 °C and 1000 mbar, with a CO_2_/N_2_ selectivity of 39 and a CO_2_/O_2_ selectivity of 42, as well as a desorption activation energy of 66.36 kJ/mol. In another study, Peng et al.^53^ developed a Mg-MOF-74/sodium alginate composite aerogel through freeze-drying, which resulted in a hierarchical porous structure. Sodium alginate created a mesoporous/macroporous network to enhance diffusion, while Mg-MOF-74 provided strong CO_2_ adsorption in the micropores. The composite with 6 wt % of Mg-MOF-74 demonstrated a CO_2_ adsorption capacity of 2.46 mmol/g at 298 K and a CO_2_/N_2_ selectivity of 35 at 25 °C and 1000 mbar, highlighting its strong potential for selective CO_2_ capture.^53^

In this work, an aluminum-based MOF, MIL-160­(Al), was incorporated into a Ca-alginate network using the direct mixing method, followed by solvent exchange and supercritical drying to prepare Ca-alginate-aerogel-MIL-160­(Al) composites. MIL-160­(Al) was selected due to its scalability, including successful tests in multikilogram scale adsorption units, and its ability to be synthesized under green conditions, such as atmospheric pressure, ensuring sustainability, and industrial relevance.^54,55^ MIL-160­(Al) with chemical formula of Al­(OH)­(O_2_C–C_4_H_2_O–CO_2_) is composed of an inorganic aluminum chain linked via five-membered ring 2,5 furandicarboxylate ligand, forming helical chains, and has a pore size ranging between 4 and 6 Å. MIL-160­(Al) provides a good selectivity for CO_2_ over N_2_ and it is stable in water and resistant to SO_2_.^56^ MOFACs synthesized in bead form were then characterized by N_2_ physisorption, Fourier transform infrared spectroscopy (FTIR), powder X-ray diffraction (PXRD), scanning electron microscopy (SEM), and thermogravimetric analysis (TGA). The performance of the MOFACs was evaluated by the CO_2_ adsorption isotherm and breakthrough curve measurements at 25 and 50 °C. The simulated gas mixture (CO_2_ 15%, N_2_ 85%) was used as the representative of the postcombustion gas. The adsorption kinetic and thermodynamic model were also described and molecular simulations were performed to study gas adsorption in the MOF and in the MOFACS.

## Experimental Section

### Materials

All chemicals were used without any further purification. Sodium salt of alginic acid (Na-alginate) from brown algae and calcium chloride (anhydrous granular, >93%) were purchased from Sigma-Aldrich. A 90% pure aluminum acetate, basic hydrate (Al­(OH)­(CH_3_COO)_2_) was purchased from Thermo Scientific. 98% pure 2,5-furandicarboxylic acid was purchased from Sikemia. Ethanol (99.9%) was obtained from Isolab. Nitrogen (99.999%) and carbon dioxide (99.999%) were purchased from air–liquid. A binary gas mixture of N_2_ and CO_2_ (15% CO_2_/85% N_2_) was purchased from Elite Gaz. Water was deionized (18 mΩ) by a water purifier device (PURELAB flex 3, ELGA Veolia).

### Synthesis of MIL-160 (Al)

MIL-160 (Al) was synthesized following a reported procedure.^57^ Al­(OH)­(CH_3_COO)_2_ (37.5 mmol, 5.85 g; Aldrich, 90%) and 2,5-furandicarboxylic acid (37.5 mmol, 6.08 g) were added to a round-bottomed flask containing 37.5 mL of distilled water and stirred for approximately 24 h. The resulting white solid was recovered by filtration, washed with ethanol, and dried under vacuum at 150 °C.

### Synthesis of AlgMIL160

Briefly, 3 g of Na-alginate was added to 100 mL of DI water and stirred at 250 rpm for 24 h (Figure S1). This was followed by adding of certain amount of MIL-160 powder to the solution which was then stirred for another 24 h at 250 rpm. 1, 3, and 9 g of MIL-160­(Al) was used to synthesize AlgMIL160 with different MIL-160 loadings of 25, 50, 75 wt %, respectively. Subsequently, the mixture containing water, Na-alginate and MIL-160­(Al) was added dropwise into 100 mL of CaCl_2_ (0.2 M) aqueous solution under constant stirring at 250 rpm. Ca^2+^ was used to trigger gelation of the alginate. As soon as the drops entered the CaCl_2_ solution, they gelled, forming spherical gel particles with diameters around 2 mm. After the completion of the dropwise addition, the spherical gel particles were filtered and solvent exchange of water with ethanol was then carried out to replace the water in the pores with ethanol, which can be extracted from the pores using supercritical CO_2_ (Figure S2). Stepwise solvent exchange was used to prevent shrinkage of beads by placing the beads into a series of mixtures of ethanol and water (25%, 50%, 75%, and last in 100% ethanol by volume). The last step performed with 100% ethanol was repeated twice. Ca-alginate-MIL-160­(Al) alcogels were then supercritically dried in an Applied Separations Speed SFE unit (Spe-ed SFE-15,000, Applied Separations, Inc.). First, the alcogel particles and a certain amount of ethanol were placed in a 25 mL tubular vessel. The vessel was then placed in the oven of the drying setup and the inlet and outlet lines were connected to the vessel. The vessel was heated to 50 °C followed by pressurizing it with CO_2_ to 90 bar. Subsequently, outlet valve was opened and adjusted to start the extraction. After 8 h, the inlet valve was closed, and the vessel was depressurized slowly to obtain dried spherical AlgMIL160 particles. Three batches of the AlgMIL160 with MIL-160­(Al) loadings of 25, 50, and 75 wt % were synthesized and named AlgMIL160-3-1, AlgMIL160-3-3, AlgMIL160-3-9, respectively. Figures 1 and S3 show the images of AlgMIL160-3–0.5 after gelation, after supercritical drying, and of prepared AlgMIL160-3-1 after supercritical drying, respectively.

**Figure 1:**
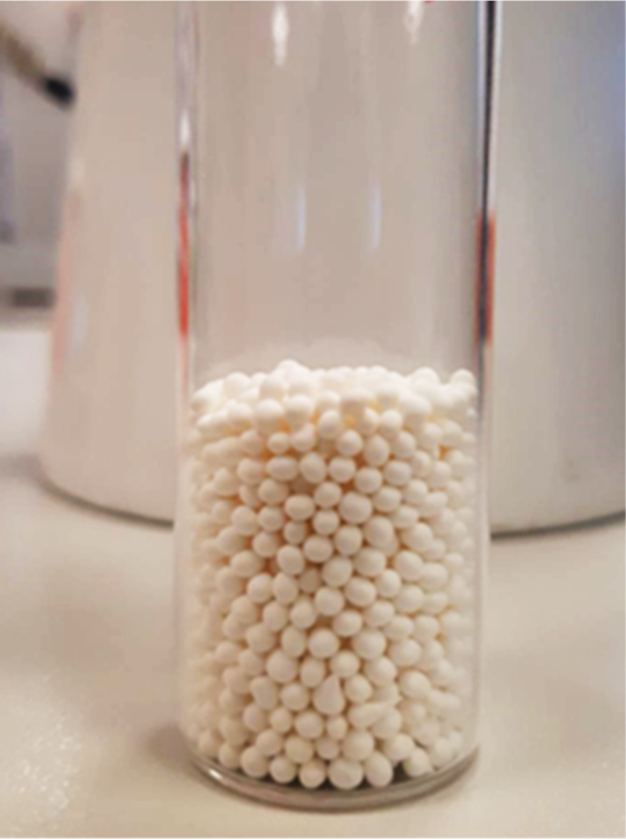
Prepared AlgMIL160-3-1 MOFACs in bead form with average particle diameter of 2.46 mm.

Performance of AlgMIL160-3-1, AlgMIL160-3-3, and AlgMIL160-3-9 beads were investigated by both static and dynamic adsorption experiments. For these batches, the Feret diameters (caliper diameter) of 100 spherical aerogel particles randomly selected from each batch were measured by using a Vernier caliper to determine the particle size distribution (PSD) and mean particle size before conducting the uptake measurements. Figure S4 illustrates the PSD of each batch, where the average particle sizes of AlgMIL160-3-1, AlgMIL160-3-3, and AlgMIL160-3-9 were determined to be 2.46, 2.17, and 2.51 mm, respectively. Subsequently, a certain number of beads were individually weighed using a balance (Mettler Toledo) to calculate the average weight of beads within each batch. The obtained average particle diameter and weight of beads were then utilized to determine the average beads’ specific volume for each batch, assuming that all aerogel particles possessed a spherical shape. The physical properties of the particles including average diameter, volume, weight, and bulk density are given in Table S1.

### Characterization

Brunauer–Emmett–Teller (BET) surface area, micropore surface area, micropore volume, pore size, and total pore volume of the synthesized MOFACs were determined by nitrogen physisorption measurement using a Micromeritics ASAP 2020 instrument. N_2_ adsorption/desorption isotherms at −196 °C were obtained within a relative pressure (*P*/*P*°) ranging from 10^–7^ to 0.995. A sample tube with a filler rod and seal frit was used. The samples were degassed at 50 °C for 1800 min prior to the analysis. Uptake amount at *P*/*P*° = 0.975 was used to determine the total pore volume of the sample. Micropore surface area and volume was obtained using the t-plot method. The desorption curve was used for determination of the average pore size through the Barrett–Joyner–Halenda (BJH) method. FTIR-ATR spectra of samples were collected at a scan rate of 64 per measurement using a Thermo Scientific iS10 FT-IR instrument. PXRD patterns were collected using an X-ray diffractometer (BRUKER D2 PHASER) with a Cu Kα source (λ = 1.5418 Å) in the 2θ range of 5–30°. Surface morphology of the samples was analyzed with a Scanning Electron Microscope (ZEISS Ultra Plus) using an accelerating voltage of 5 kV. SEM/EDX was used to identify the presence of aluminum in the MOF particles dispersed in the aerogel network. Static gas adsorption isotherms via volumetric gas sorption analysis were collected at 25 °C within a pressure range of 0 to 1000 mbar. Micromeritics 3Flex instrument was used for static CO_2_ and N_2_ sorption isotherms. Prior to each analysis, all of the tested adsorbents were degassed at 50 °C for 4 h. The obtained isotherm data of each composite was fitted to single site Langmuir model. Furthermore, ideal adsorption solution theory (IAST)^58^ was used to predict binary 15% CO_2_/85% N_2_ adsorption isotherms and CO_2_/N_2_ selectivity.

### Molecular Simulations and Modeling

The CO_2_ and N_2_ adsorption properties of MIL-160­(Al) were computed at 298 K and pressures in between 0 and 1000 mbar through Grand Canonical Monte Carlo (GCMC) simulations utilizing the RASPA software.^59^ First, the crystallographic information file for MIL-160­(Al), PIBZUY03, was acquired from the Cambridge Structural Database,^60^ which archives experimentally reported MOFs. The corresponding structure was then optimized using the Forcite module within the Materials Studio software,^61^ converting its symmetry to the *P*1 space group. Density functional theory (DFT)-level quality charges, calculated using the partial atomic charge Predicter for Porous Materials based on Graph Convolutional Neural Network (PACMAN) software,^62^ were used to assign the partial charges of the MOF. A cutoff of 12.8 Å was employed, leading to an expansion of the MOF’s unit cell to a 2 × 2 × 3 configuration within the simulation box. GCMC simulations involved 50,000 initialization cycles followed by 50,000 production cycles for ensemble averaging. For the CO_2_ and N_2_ molecules, translation, rotation, reinsertion, regrow, and swap moves were implemented in the simulations. The interactions between the MOF atoms with CO_2_ and N_2_ molecules, as well as the interactions between CO_2_ and N_2_ molecules, were modeled using Lennard–Jones 12-6 potentials and Coulombic potentials, with the framework atoms being described by the DREIDING force field.^63^ CO_2_ molecules were represented using TraPPE potentials and were modeled as a rigid, linear molecule with three sites, each containing partial point charges at their respective centers.^64^ N_2_ molecules were defined as a three-site rigid molecule with N atoms at the two sites, and the third site was the center of mass with partial charges.^65^ Electrostatic interactions were computed using the Ewald summation method.^66^ As a result, single-component gas adsorption data for MIL-160­(Al) were obtained from these molecular simulations. We also proposed a simple model to predict both the CO_2_ and N_2_ adsorption capacities of the composites synthesized at different weight percentages, utilizing the gas adsorption data obtained from molecular simulations of MIL-160­(Al) and experimentally measured gas adsorption data of alginate. The model is given by

$$composite^{\prime }s\;uptake=\frac{\left({N_{i}}_{Sim-MIL-160\left(Al\right)}\times m_{MIL-160\left(Al\right)}\right)+\left({N_{i}}_{\mathrm{Exp}-Alg}\times m_{Alg}\right)}{m_{AlgMIL160}}$$

where *N*_*i*_ denotes the CO_2_ and N_2_ adsorption amount measured either through simulation or experiments for the respective materials, while *m*_MIL‑160(Al)_ and *m*_Alg_ represent the masses of MIL-160­(Al) and alginate used in the synthesis of the composite. The term *m*_AlgMIL160_ refers to the total mass of the composite.

### Dynamic Adsorption Measurements

The schematic of the dynamic adsorption experimental setup used for the CO_2_ adsorption experiments is shown in Figure 2. A stainless-steel tube with an internal diameter of 1.02 cm was used as the adsorption column. Temperature of the bed was controlled by flowing water through a silicon tube wrapped around the bed. The tube was connected to a heating water circulator (Cole Parmer, model 12,108-15). Mass flow controllers (MFC) including ALICAT MC-50SCCM-D and Teledyne Hastings HFC202 were used to regulate the volumetric flow rates of the 15% CO_2_/85% N_2_ gas mixture and the purging gas, N_2_, respectively. The flow rate of the CO_2_/N_2_ gas mixture was kept constant at 5 N mL/min. A CO_2_ sensor (GasLab, SprintIR-W 100% CO_2_ sensor) was placed on the outlet line to measure the concentration of CO_2_ continuously. Prior to the adsorption experiments, the breakthrough curve of the empty bed without any loaded material was obtained to take into account the lag time effect due to dead volume between the MFC and the sensor in the uptake calculations. For each experiment, the bed was packed with AlgMIL160 beads. Afterward, the bed was purged for 4 h with N_2_ at a constant temperature of 50 °C. Following the purging step, the 15% CO_2_/85% N_2_ mixture was introduced to the bed, and the measurement of CO_2_ concentration in the outlet gas was started. The experiment was continued until the exit concentration reached the inlet concentration. The integration of the breakthrough curves enabled the determination of the CO_2_ uptake of MOFACs from a binary gas mixture of 15% CO_2_/85% N_2_ in dynamic mode. The physical properties of the columns and uptake calculations are given in the Supporting Information Table S2 and Section 4, respectively.

**Figure 2:**
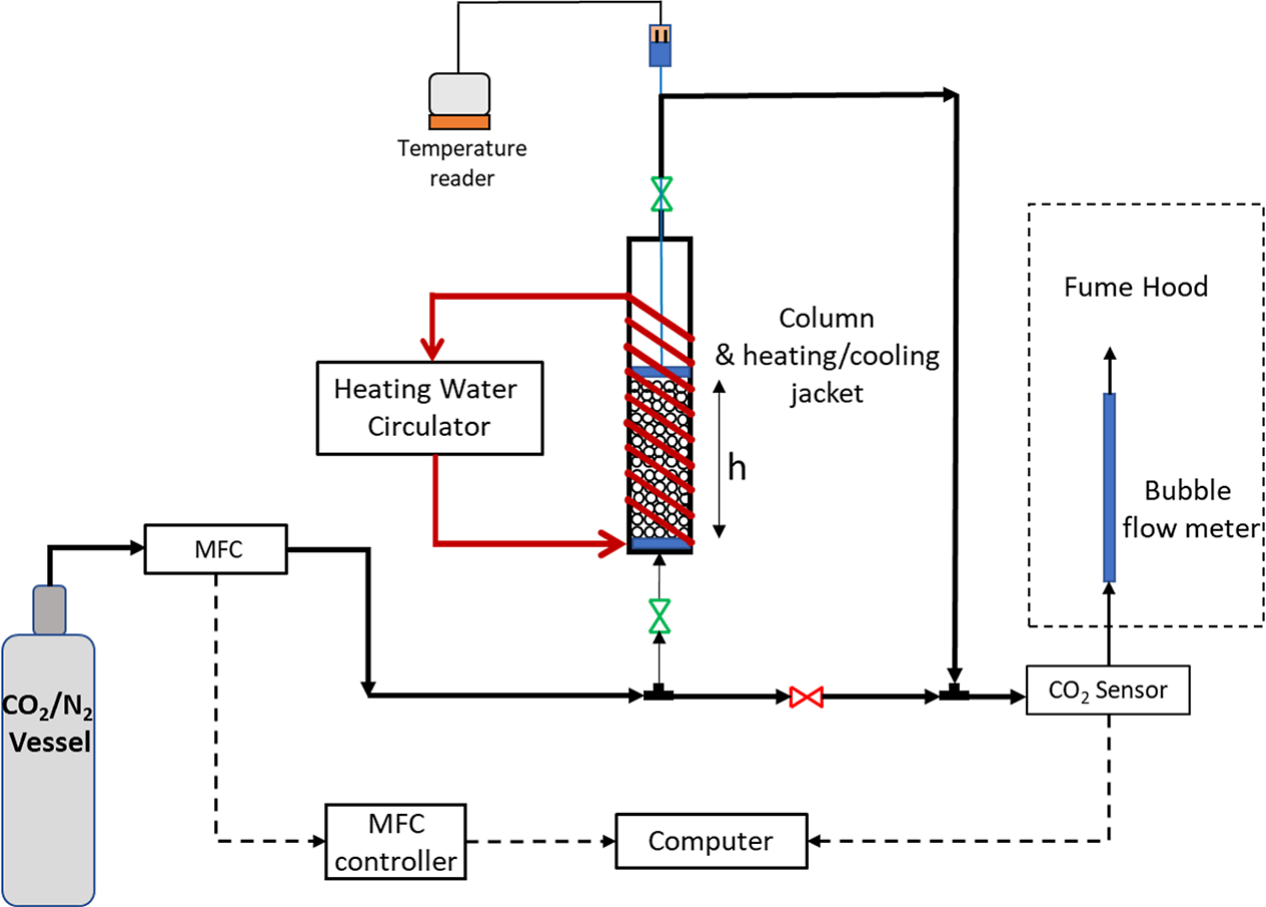
Dynamic adsorption experimental setup.

## Results and Discussion

N_2_ physisorption analysis was performed to determine the textural properties of the samples. The adsorption and desorption isotherms of Ca-alginate, MIL-160­(Al), AlgMIL160-3-1, AlgMIL160-3-3, and AlgMIL160-3-9 are given in Figure 3. The BET surface area values and micropore area and volume given in Table 1 showed that the incorporation of the MOFs into the polymeric network of the aerogel was successfully carried out without blocking the pores of the MOF. It was observed that the BET surface area increased from 556 to 768 m^2^/g by decreasing the initial mass ratio of Ca-alginate/MIL-160­(Al) from 3/1 to 3/9. The BET surface areas of pure Ca-alginate and MIL-160­(Al) were around 252 and 1218 m^2^/g, respectively, showing that the increase in the surface area of AlgMIL160­(Al) with increasing MIL-160­(Al) fraction is due to the presence of MOF particles with open pore cages in the nanocomposite. Furthermore, there was an initial sharp increase in the adsorption isotherm along with a hysteresis during desorption, which show the presence of micromeso-and macro pore structure in MOFACs in line with the nature of the composite built by combination of aerogel and MOF structures.

**Figure 3:**
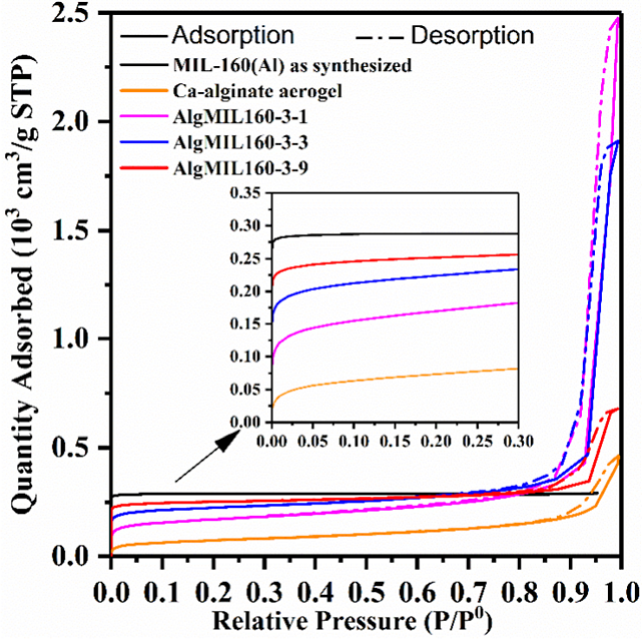
N_2_ adsorption–desorption isotherms for Ca-alginate, MIL-160­(Al), AlgMIL160-3-1, AlgMIL160-3-3, AlgMIL160-3-9 at −196 °C.

**Table 1:** Pore Properties of the Samples

sample	initial Alg g	initial MOF g	BET surface area m^2^/g	total pore volume cm^3^/g	micropore volume cm^3^/g	micropore area m^2^/g	external surface area m^2^/g
AlgMIL160-3-9	3.00	9.00	768	1.04	0.35	667	102
AlgMIL160-3-3	3.00	3.00	706	2.94	0.25	475	230
AlgMIL160-3-1	3.00	1.00	556	3.81	0.14	264	292
MIL-160(Al)			1218	0.45	0.45	1208	10
Ca-alginate			252	0.71	0.02	36	216

SEM images of pure MIL-160­(Al) and the cross section of MOFAC beads were captured using an accelerating voltage of 5 kV (Figure 4 for AlgMIL160-3-9 and Figure S5 for AlgMIL160-3-1 and AlgMIL160-3-3). These images of the synthesized MOFACs with different MOF loadings show an interconnected porous network of the aerogel where the MOF particles are equally dispersed along the network which confirms that MIL-160­(Al) was incorporated in to the aerogel network successfully. By increasing the MOF loading, the number of MOF particles increases as expected. Also, the crystalline MOF particles (Figure 4a) were observed in amplified SEM images (Figures 4b,c, S5b and S5d) showing the structure of MIL-160­(Al) was preserved during the synthesis. SEM–EDX analysis was also conducted on these crystalline particles to confirm the presence of Al in the crystalline phase of the composites since MIL-160­(Al) is an aluminum-based MOF (Figure S6). One point from a randomly selected particle along with *a* point through the aerogel network was analyzed. The result shows the presence of the aluminum in the target particles showing that those particles are the initial aluminum-based MOF particles that were incorporated into the alginate solution during the synthesis while there was no sign of Al in the network.

**Figure 4:**
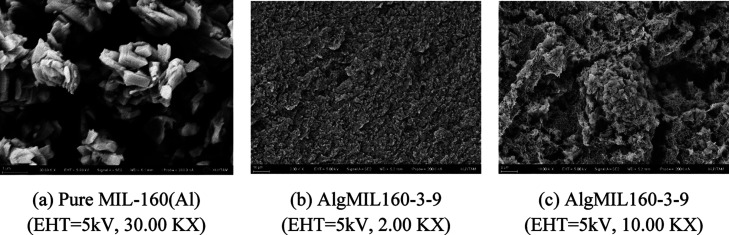
SEM images of (a) pure MIL-160­(Al) powder with 30 KX magnification, (b) AlgMIL160-3-9 with 2KX magnification, (c) AlgMIL160-3-9 with 10 KX magnification.

PXRD patterns of the synthesized MOFACs were obtained for the samples with different MIL-160­(Al) loadings and compared with the pattern of the pure MOF. The PXRD pattern of each MOFAC was normalized to the first diffraction peak at 2θ = 8.45° which had the maximum intensity in all patterns. The PXRD pattern of MIL-160­(Al) shows the characteristic peaks of MIL-160­(Al).^57,67–69^ Figure 5a shows that the crystalline structure of the MOF was well preserved during the synthesis of the MOFACs (AlgMIL160-3-1, AlgMIL160-3-3, and AlgMIL160-3-9) as the obtained diffraction peaks matched well with the characteristic peaks of MIL-160­(Al). FTIR spectra of Ca-alginate, MIL-160­(Al), and AlgMIL160 with different MIL-160­(Al) loadings are shown in Figure 5b. The vibration bands associated with the C–O–C and COO^–^ of the aerogel network were observed in AlgMIL160 IR spectra. IR bands appearing at 1582, 1417, 1402, 1367, 1241, 991, 971, 823, 807, and 783 cm^–1^ marked by star signs were assigned to MIL-160­(Al). Silva et al.^69^ and Wahiduzzaman et al.^70^ attributed the band at 1582 to the stretching vibration of the C=C bond in the furan ring. However, it was assigned to asymmetric vibration of COO^–^ group in 2,5 furandicarboxylate in case of hydrated MIL-160­(Al) in a study conducted by Henry and Samokhvalov.^71^ It should be mentioned that they also observed the appearance of a new vibration peak at 1576 cm^–1^ after activation of the MOF which was assigned to the C=C bond in the furan ring. The band at 1417 cm^–1^ can be assigned to the stretching vibration of the wagging vibration of the –CH– group,^69^ while it is also reported that the bands at 1417 and 1402 can be attributed to the symmetric vibration of COO^–^ group in 2,5 furandicarboxylate.^70,71^ Also, the bands appearing between 1000 and 1400 cm^–1^ corresponded to the stretching vibrations of the furan rings. The C–H out-of-plane bending vibrations of the furan ring were observed at 783 cm^–1^.^69^ FTIR analysis confirmed that the synthesized MIL-160­(Al) was free from an unreacted linker. The intensity of peaks changed in line with MIL-160­(Al) loading, where the sample with higher MIL-160­(Al) loading showed more intense vibration peaks.

**Figure 5:**
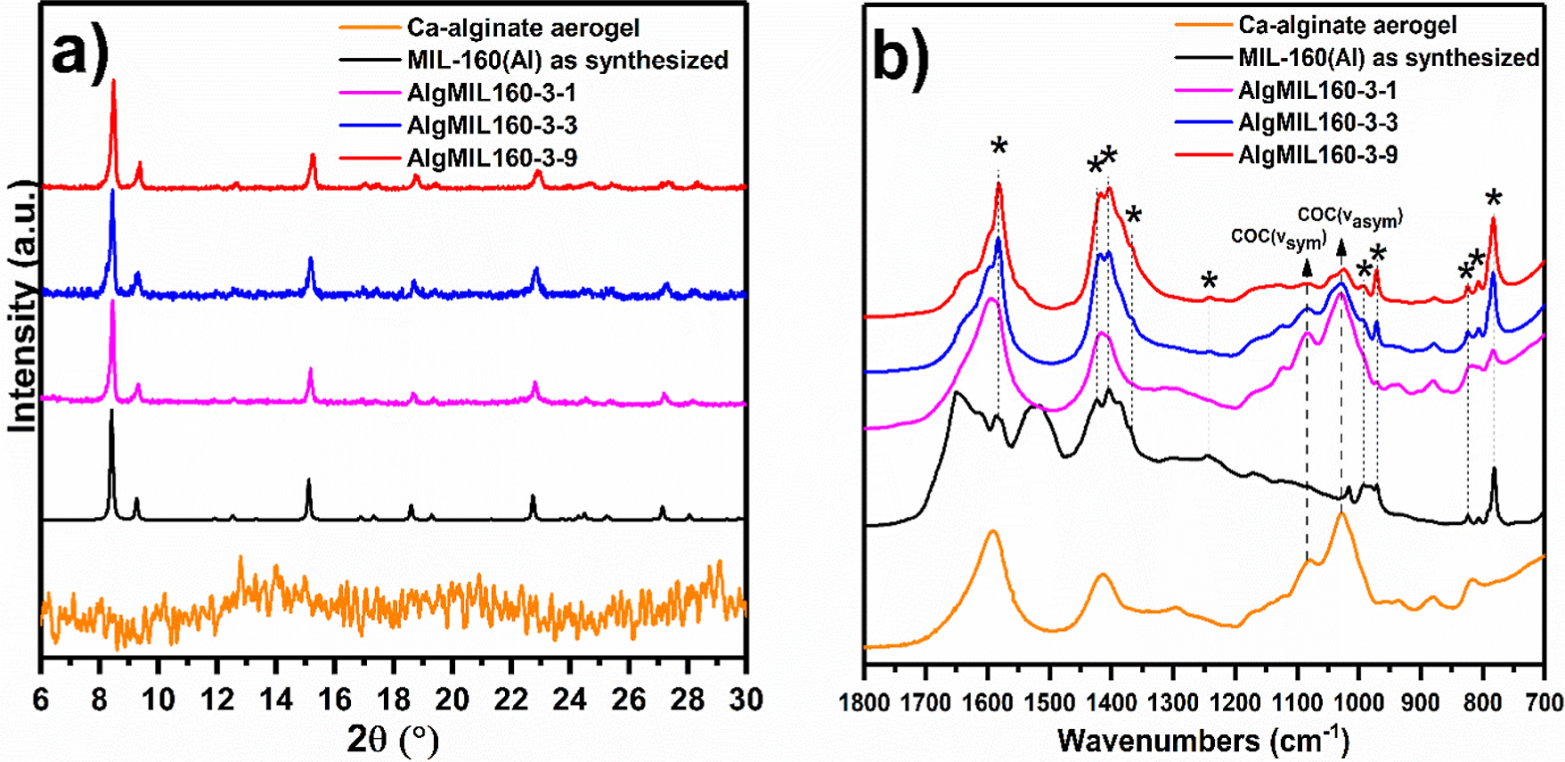
(a) PXRD patterns and (b) FTIR spectra of pure MIL-160­(Al) and AlgMIL160 with different MIL-160­(Al) loadings.

TGA curves shown in Figure 6 revealed the first weight loss of AlgMIL160 started around 50 °C and lasted up to 200 °C. Interestingly, thermal stability of the AlgMIL160 was higher than both pure MIL-160­(Al) and pure Ca-alginate at this temperature range, making the new nanocomposites better adsorbent candidates than pure MIL-160­(Al). Furthermore, the weight loss at this temperature range increased by increasing the MIL-160­(Al) content of the AlgMIL160. AlgMIL160-3-1 showed a weight loss of around 20% as the temperature reached 200 °C. However, the MOFACs with higher MIL-160­(Al) content had higher weight loss at temperatures below 200 °C as pure MIL-160­(Al) showed more than 25% weight loss when it was heated up to 100 °C. A sharp decrease of the weight was observed for AlgMIL160 starting with a fast decomposition of the material when temperature increased to above 200 °C, a similar behavior to that of pure Ca-alginate. Comparison with the pure MIL-160­(Al) and pure Ca-alginate reveals that this weight loss at temperature range of 200–350 °C is due to the decomposition of Ca-alginate component of the AlgMIL160. Finally, the weight percentage stabilized at 20% at temperatures above 450 °C.

**Figure 6:**
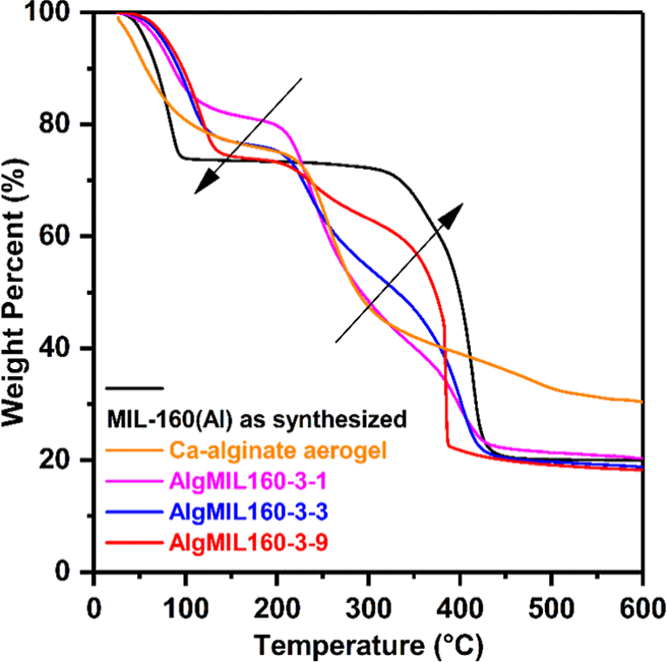
TGA curves of pure MIL-160­(Al) and MOFACs with different MOF loadings.

Both the experimentally measured and simulated single-component adsorption isotherms of CO_2_ on pure MIL-160­(Al) and AlgMIL160 at 25 °C with an equilibrium pressure range of 0–1000 mbar are depicted in Figure 7a,b, respectively. CO_2_ uptake of MIL-160­(Al) increased up to 3.97 mmol/g when the equilibrium pressure reached 1000 mbar, while AlgMIL160-3-1, AlgMIL160-3-3, and AlgMIL160-3-9 showed an uptake of 1.61, 2.29, and 3.19 mmol/g at 1000 mbar, respectively. There is good agreement between the experimentally measured adsorption isotherm and the simulated one in Figure 7a. For instance, at a pressure of 1000 mbar and a temperature of 25 °C, the simulated CO_2_ uptake of MIL-160­(Al) was 4.32 mmol/g, which closely aligns with the experimentally measured value of 4.11 mmol/g. Figure 7b presents a comparative analysis of the experimentally measured CO_2_ uptakes of AlgMIL160 composites with those predicted by our theoretical model which integrates simulated CO_2_ uptakes of pure MIL-160­(Al) with the experimentally measured CO_2_ uptakes of pure Ca-alginate. The results reveal a good agreement between the model predictions and the experimental data, affirming the model’s precision in predicting gas adsorption across different pressure levels.

**Figure 7:**
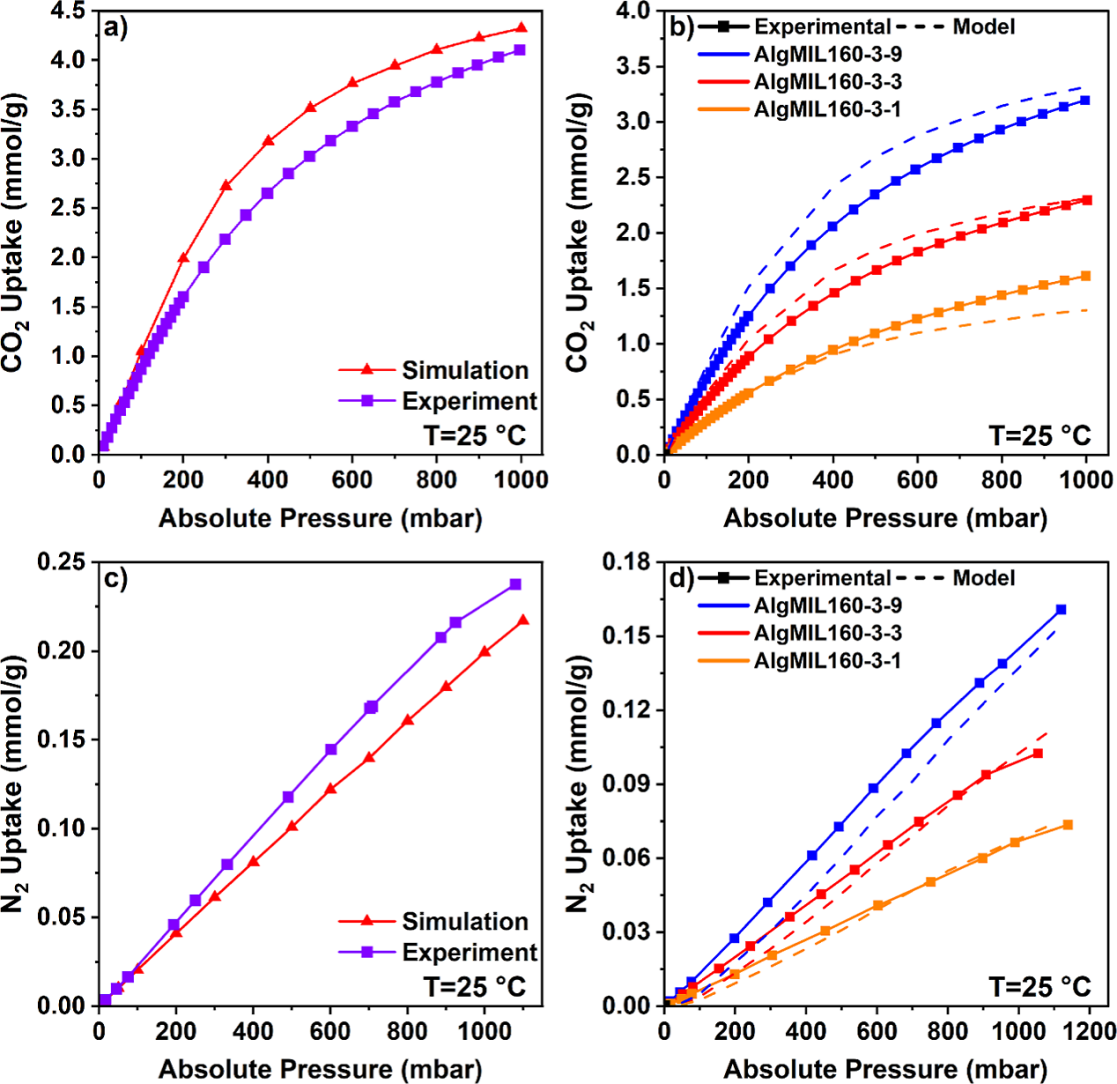
(a) Simulated and experimental CO_2_ adsorption isotherms for pure MIL-160­(Al) at 25 °C over a pressure range of 0–1000 mbar. (b) Comparison of experimentally measured CO_2_ uptake and model-predicted values for the AlgMIL-160 composite series at 25 °C over a pressure range of 0–1000 mbar. (c) Simulated and experimental N_2_ adsorption isotherms for pure MIL-160­(Al) at 25 °C over a pressure range of 0–1200 mbar. (d) Comparison of experimentally measured N_2_ uptake and model-predicted values for the AlgMIL160 composite series at 25 °C over a pressure range of 0–1200 mbar.

Within the composite series, AlgMIL-160-3-9 demonstrated the highest CO_2_ uptake capacity; both experimental data and model predictions show consistent results, particularly under low-pressure conditions. Similarly, for AlgMIL-160-3-3, the model accurately predicts a CO_2_ uptake of 2.31 mmol/g, closely matching the experimentally observed value of 2.29 mmol/g at 1000 mbar and 25 °C. However, for AlgMIL-160-3-1, while the model performs reliably up to a pressure of 500 mbar, it tends to underpredict CO_2_ uptake at higher pressures compared to the experimental results. This can be attributed to enhanced interactions between CO_2_ molecules and the composite material that are not accounted for in the approximate model. The Ca-alginate matrix might facilitate additional CO_2_ adsorption through physical or chemical interactions such as induced dipole moments or specific binding sites that become more prominent at higher pressures.

The experimentally measured and simulated single-component adsorption isotherms of N_2_ on pure MIL-160­(Al) demonstrate good agreement, as illustrated in Figure 7c. At 298 K and 0.9 bar, the simulated N_2_ uptake was 0.18 mmol/g, closely aligning with the experimental value of 0.21 mmol/g. Our model effectively captures the N_2_ adsorption performance across all AlgMIL160 composites, as depicted in Figure 7d. Specifically, at 298 K and 0.9 bar, the model predicts N_2_ uptakes of 0.123, 0.092 and 0.062 mmol/g for AlgMIL-160-3-9, AlgMIL-160-3-3, and AlgMIL-160-3-1, respectively. These predictions closely match the corresponding experimental values of 0.131, 0.09, and 0.060 mmol/g, demonstrating the model’s accuracy and reliability. These findings underscore the effectiveness of combining simulation data with experimental measurements of pure components to guide the design of optimized composite materials.

The equilibrium uptake of N_2_ at 1000 mbar was much lower than those of CO_2_ uptake varying from 0.06 to 0.13 mmol N_2_/g_adsorbent_ with increasing the Ca-alginate/MIL-160­(Al) ratio from 3/1 to 3/9, respectively. A Langmuir single-site adsorption model was fitted into the obtained experimental isotherm data (Figure 8) and the isotherm parameters were reported in Table S3. The fitted parameters were used in the IAST model to predict the binary adsorption isotherms (Figure 9) and CO_2_/N_2_ selectivity (Figure S7) for 15% CO_2_/85% N_2_ gas mixture on the MOFACs. Comparison of the IAST binary isotherms for a gas mixture of 15% CO_2_/85% N_2_ with those of experimental corresponding single-component ones revealed very close uptake values of both CO_2_ and N_2_. For example, in the case of AlgMIL160-3-9, the experimental isotherm of single-component CO_2_ adsorption at 150 mbar and its corresponding binary IAST model of the 15%CO_2_–85%N_2_ mixture at 1000 mbar showed similar uptakes of 0.98 and 0.97 mmol/g, respectively (Table S4). The CO_2_/N_2_ selectivity calculated from the IAST model for AlgMIL160-3-1, AlgMIL160-3-3, and AlgMIL160-3-9 composites increased by increasing the pressure varying in the ranges of 53.2–61.4, 58.2–70.7, and 57.7–69.7, respectively. Increasing the initial MOF content from 25 to 50 wt % in the MOFACs resulted in an increase in CO_2_/N_2_ IAST selectivity. However, further increases in the MOF content led to a slight decrease in selectivity, suggesting that, at higher MOF contents, the growth in N_2_ uptake exceeds that of CO_2_ uptake.

**Figure 8:**
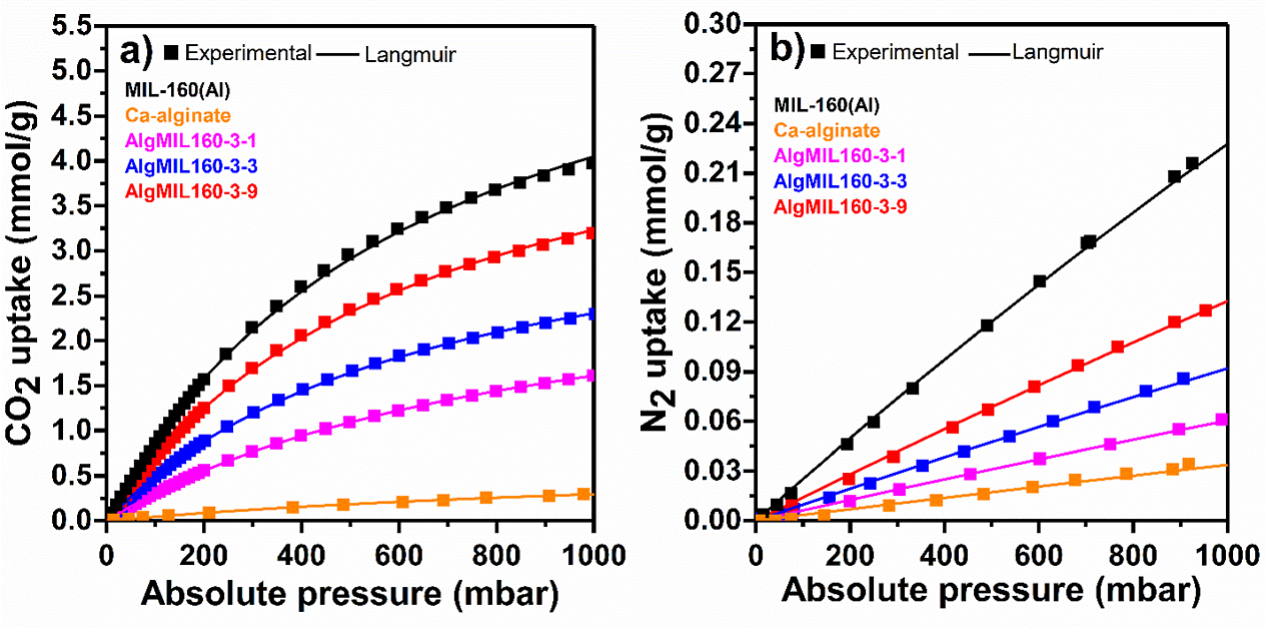
(a) CO_2_ and (b) N_2_ single component adsorption isotherms for pure MIL-160­(Al) and AlgMIL160 composites at 25 °C at pressure range of 0–1000 mbar.

**Figure 9:**
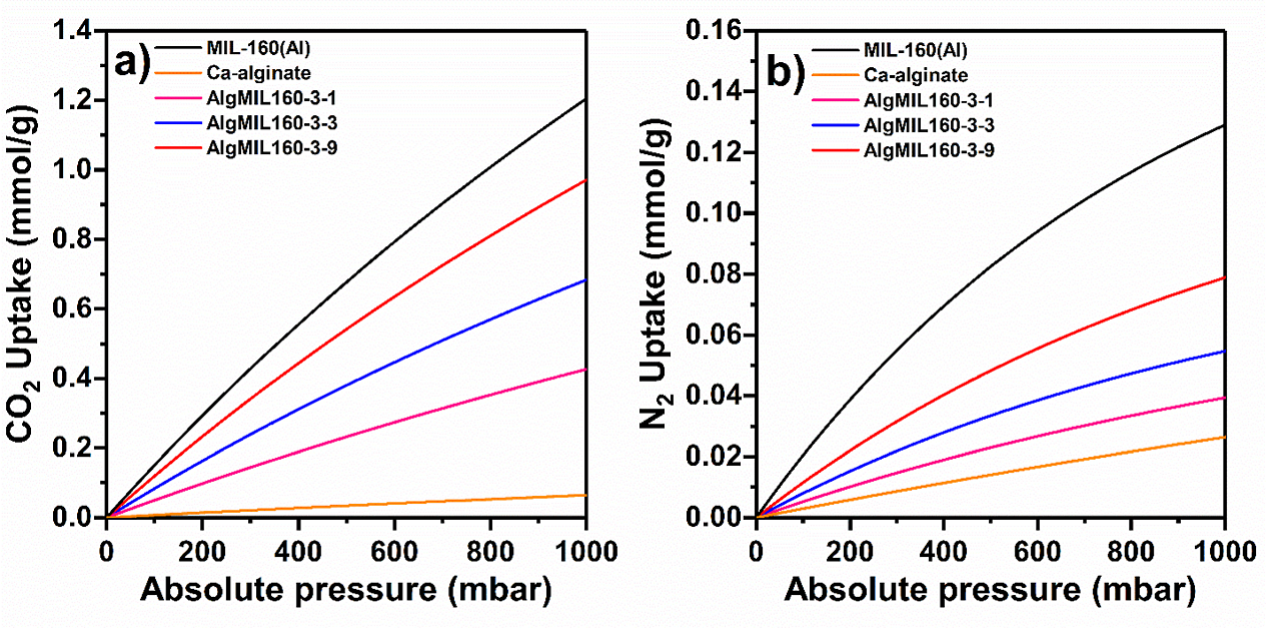
(a) CO_2_ and (b) N_2_ binary adsorption isotherms obtained by IAST at 25 °C for a gas mixture of 15% CO_2_/85% N_2_.

The dynamic adsorption experiments were carried out to obtain the breakthrough curves for the prepared MOFACs with different MOF loadings at 25 and 50 °C and partial pressure of 150 mbar for CO_2_ (Figures 10 and S8a–e). It should be noted that the breakthrough of the empty bed (shown in dashed orange-colored lines) was also obtained to take into account the effect of the lag time of gas flow between the sensor and the MFC on the response time of the CO_2_ sensor, which was necessary in the uptake calculations. The lag in the response time was due to the time that takes the gas stream feeding from the MFC to reach the sensor and also the effect of axial dispersion of CO_2_ along the lines and column. Increasing the MIL-160­(Al) content in the AlgMIL160 composites resulted in longer breakthrough times due to the increasing equilibrium uptake capacity of the MOFACs. The equilibrium CO_2_ uptake for these MOFACs were calculated using eq 1 given in the Supporting Information and the results were summarized in Table S4. Since the experimental dynamic breakthrough measurements were conducted using a gas mixture of 15% CO_2_/85% N_2_, the equilibrium uptake results of these experiments were compared with the equilibrium uptakes calculated by the binary IAST isotherms for the 15% CO_2_/85% N_2_ gas mixture at 1000 mbar (Figures 8, S9, and Table S4). The single-component CO_2_ adsorption isotherms for AlgMIL160-3-1, AlgMIL160-3-3, and AlgMIL160-3-9 demonstrated CO_2_ uptakes of 0.43, 0.70, and 0.98 mmol/g, respectively. These values surpassed the CO_2_ uptake of pure MOF (3.97 mmol/g) when accounting for the MOF content in the composites, highlighting the synergistic interaction between the aerogel and the MOF in the composite material. For instance, in the MOFAC composite AlgMIL160-3-9 with a 75 wt % MOF loading, the expected single-component CO_2_ uptake at 150 mbar was 0.92 mmol/g (equivalent to 75% of the pure MOF’s CO_2_ uptake of 1.24 mmol/g at the same pressure), while the measured CO_2_ uptake was 0.98 mmol/g. Furthermore, the IAST isotherms was calculated showing the equilibrium uptake of CO_2_ for AlgMIL160-3-1, AlgMIL160-3-3, and AlgMIL160-3-9 at 1000 mbar were 0.43, 0.68, and 0.97 mmol/g, while their corresponding values in dynamic measurements were 0.44, 0.55, and 0.86 mmol/g, respectively. The slightly reduced uptake could be attributed to competitive adsorption. The experiments at 25 °C were repeated three times named as cycle 1, 2, and 3 to investigate the performance of the materials in a series of adsorption tests (Figure S8a–c). Successive breakthrough runs were conducted at 25 °C while after each run, the bed was regenerated by passing pure N_2_. The performances of all types of AlgMIL160 composites were promising as there were no changes in breakthrough curves after 3 cycles of adsorption tests at the same operation conditions. Figure S8d,e show that the breakthrough curve of the experiments conducted at 50 °C reached equilibrium faster than those carried out at 25 °C showing a decrease in CO_2_ uptake by increasing temperature due to the exothermic nature of the adsorption on these materials. Furthermore, a comparison of the breakthrough curves of AlgMIL160-3-9 with pure MIL-160­(Al) in powder form at 25 °C (Figure S10) revealed that the Ca-alginate–MIL-160­(Al) composite exhibited higher CO_2_ uptake relative to the MOF loading in the composite. This enhancement can be attributed to reduced mass transfer limitations in the composite form, as well as the synergistic interaction between Ca-alginate and MIL-160­(Al) within the composite structure. Sharper breakthrough curve of the composites also demonstrates the advantages of these systems due to shorter cycle times.

**Figure 10:**
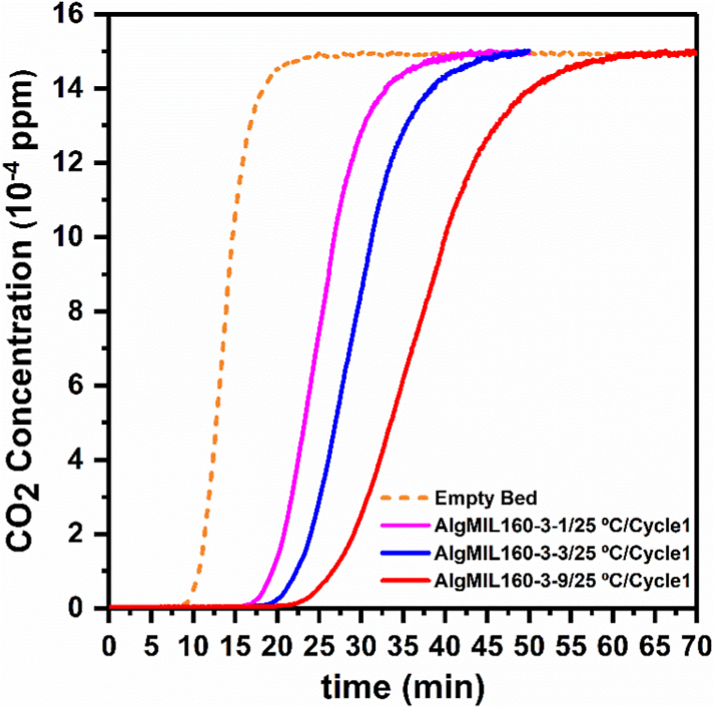
Breakthrough curves for AlgMIL160-3-1, AlgMIL160-3-3, AlgMIL160-3-9 at 25 °C.

## Conclusions

The development of MOFACs in bead form addresses common challenges in CO_2_ capture by MOFs while leveraging the synergistic interaction between the aerogel and MOF in the composite, resulting in enhanced CO_2_ uptake by MIL-160­(Al). In this study, Ca-alginate-MIL-160­(Al) MOFACs (AlgMIL160) were synthesized using sol/gel and direct mixing methods, followed by supercritical drying. Characterization by nitrogen physisorption, PXRD, FTIR, and SEM confirmed the successful incorporation of MOFs into the aerogel network while maintaining their structure. Adsorption tests in both static and dynamic modes were performed, and the resulting isotherms were fit to the Langmuir and IAST models. The single-component gas adsorption isotherms demonstrated that the MOFACs have higher CO_2_ uptake per unit weight than the pure MOF, indicating the complementary properties of the aerogel and MOF. Incorporating MOFs into the aerogel enhanced the CO_2_/N_2_ selectivity at 25 °C and 1000 mbar, increasing from 53 to 70 with 75% MOF loading. Both experimental and simulated CO_2_ adsorption isotherms showed strong agreement with the model accurately predicting performance at higher pressures. In dynamic adsorption with a binary mixture of 15% CO_2_/85% N_2_, the MOFAC performance was close to that of single-component CO_2_ adsorption, with a slight reduction due to competitive adsorption between CO_2_ and N_2_.

## Supplementary Material



## Notes

Preparation of MOFACs; PSD of nanocomposite beads; SEM/EDX; dynamic uptake calculations; adsorption isotherm parameters; and breakthrough curves (PDF)
