# Supplementary material for: Calcium Alginate Aerogel-MIL160 Nanocomposites for CO2 Removal
Source: Langmuir. 2025 May 8;41(19):11912–22. doi: 10.1021/acs.langmuir.5c00143 (PMC12550840; doi:10.1021/acs.langmuir.5c00143)
Supplement: Supplementary file 1 [file la5c00143_si_001.pdf]

Supporting Information:

Calcium Alginate Aerogel-MIL160 Nanocomposites for CO_2 Removal

Hamed Yousefzadeh[a,b], Aysu Yurdusen[c], Ayça Tüter[b], Gokhan O. Aksu[b], Georges Mouchaham[c], Seda Keskin[b,d], Christian Serre[c], Can Erkey[b,d,e]*

[a] Department of Chemical Engineering, Yeditepe University, Atasehir, Istanbul 34755, Türkiye

[b] Department of Chemical and Biological Engineering, Koç University, Rumelifeneri Yolu, Sariyer, Istanbul 34450, Türkiye

[c] Institut des Matériaux Poreux de Paris, ESPCI Paris, Ecole Normale Supérieure, CNRS, PSL University, Paris 75005, France

[d] Koç University TÜPRAŞ Energy Center (KUTEM), Koç University, Rumelifeneri Yolu, Sariyer, Istanbul 34450, Türkiye

[e] Koç University Hydrogen Technologies Center (KUHyTech), Koç University, Rumelifeneri Yolu, Sariyer, Istanbul 34450, Türkiye

*Corresponding Author: cerkey@ku.edu.tr

1. Preparation of MOFACs

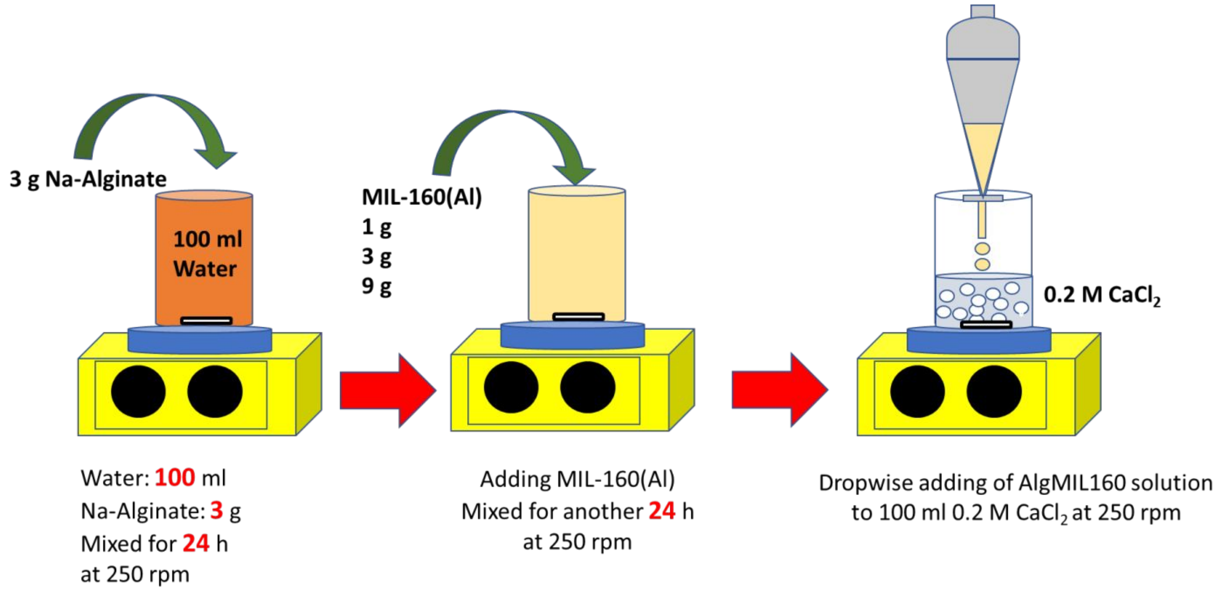

Figure S1. Synthesis steps of AlgMIL160 hydrogels.

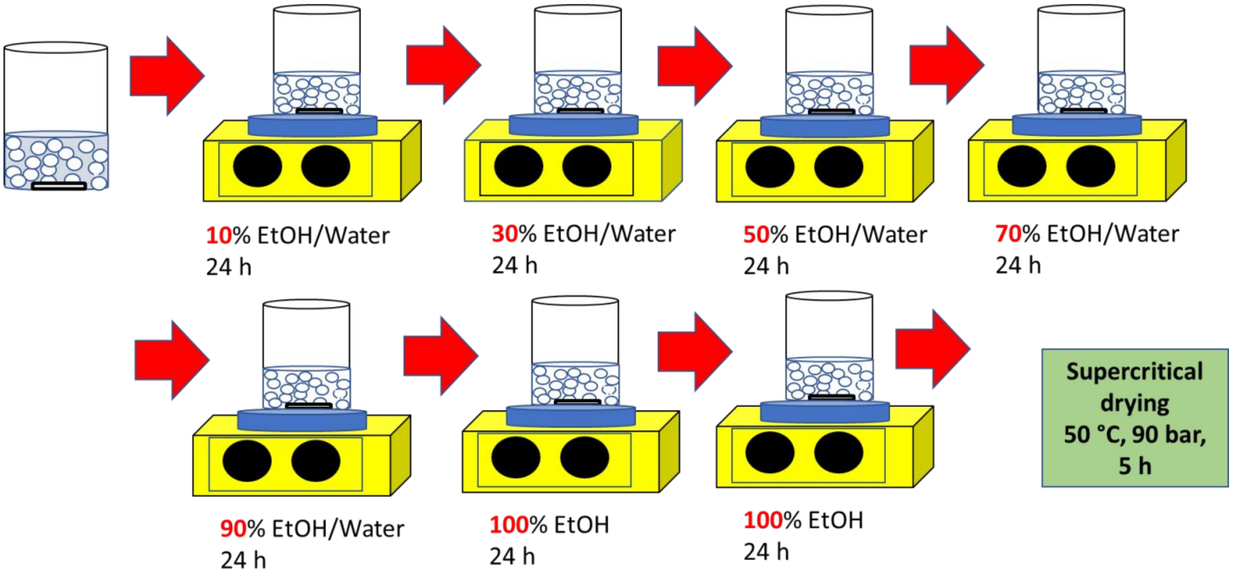

Figure S2. Stepwise solvent exchange of MOFAC hydrogels with ethanol followed by supercritical drying.

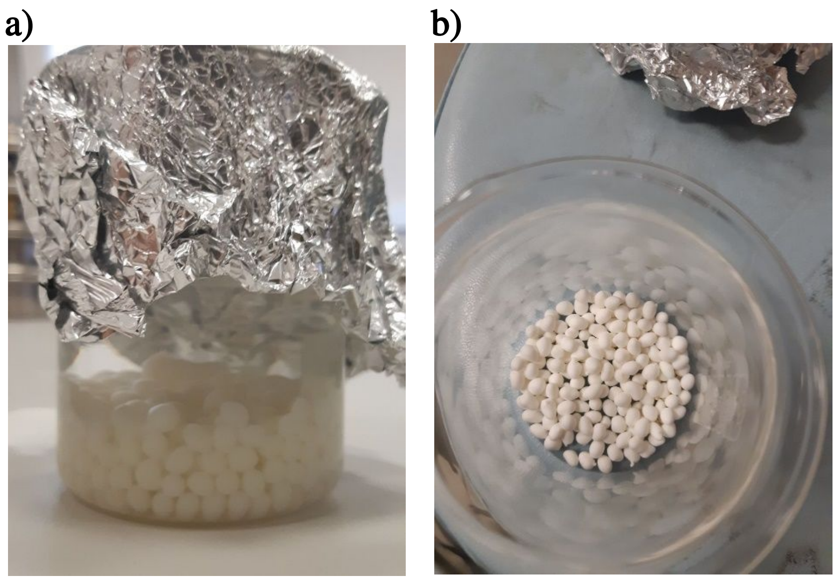

Figure S3. Images of AlgMIL160 composites (a) AlgMIL160-3-0.5 (After Gelation), (b) AlgMIL160-3-0.5 (After Drying)

2. Particle size distribution of MOFAC particles for batches with different MOF loadings

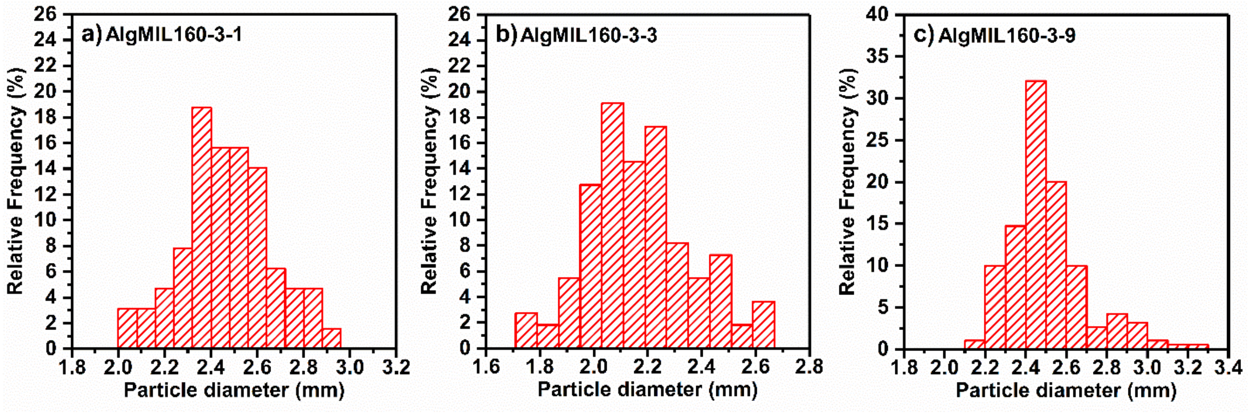

Figure S4. Particle size distribution for prepared MOFACs. (a) AlgMIL160-3-1, (b) AlgMIL160-3-3, (c) AlgMIL160-3-9.

Table S1. Physical properties of the synthesized MOFACs.

Sample	Particle diameter*	Particle volume*	Particle weight*	Bulk density
	mm	cm^3	g	g/cm^3
AlgMIL160-3-1	2.46	0.0078	0.0010	0.1284
AlgMIL160-3-3	2.17	0.0053	0.0016	0.2992
AlgMIL160-3-9	2.51	0.0083	0.0032	0.3867

*The values represent the average of 100 particles randomly characterized.

Table S2. Physical properties of adsorption beds filled with different MOFACs.

sample	Bed Length	Bed ID	Particle diameter	Adsorbent amount (before tests)	Adsorbent amount (after tests)
	cm	cm	mm	g	g
AlgMIL160-3-1	15	1.022	2.46	1.0	0.81
AlgMIL160-3-3	10	1.022	2.17	1.0	0.89
AlgMIL160-3-9	7	1.022	2.51	1.0	0.86

3. SEM/EXD images and N_2 physisorption analysis of the composites

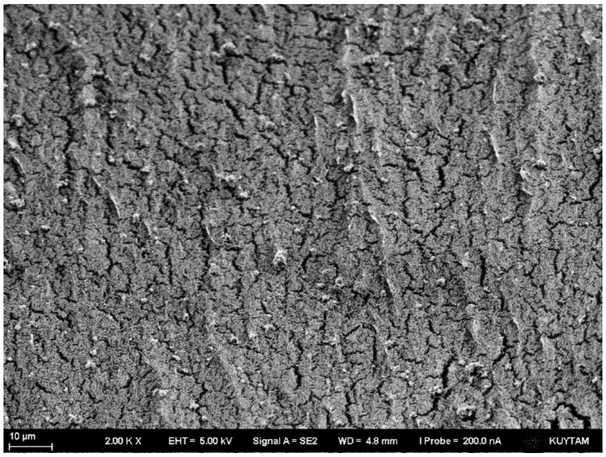

(a) AlgMIL160-3-1 (EHT=5kV, 2.00 KX)

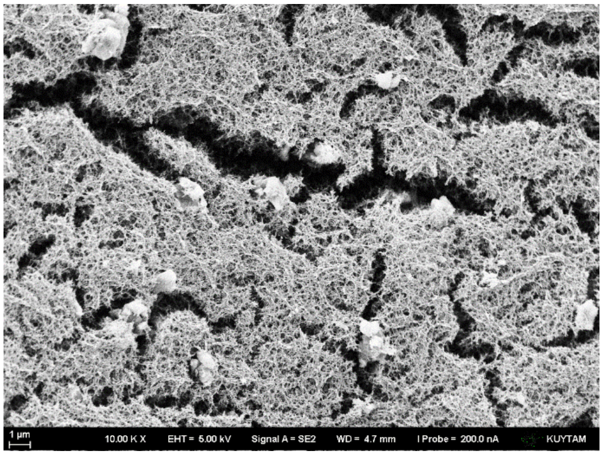

(b) AlgMIL160-3-1 (EHT=5kV, 10.00 KX)

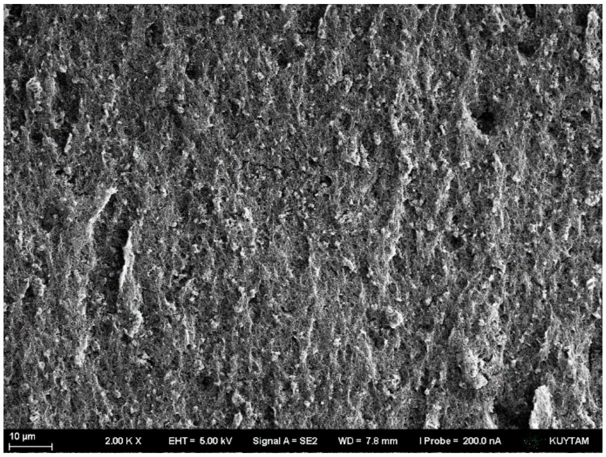

(c) AlgMIL160-3-3 (EHT=5kV, 2.00 KX)

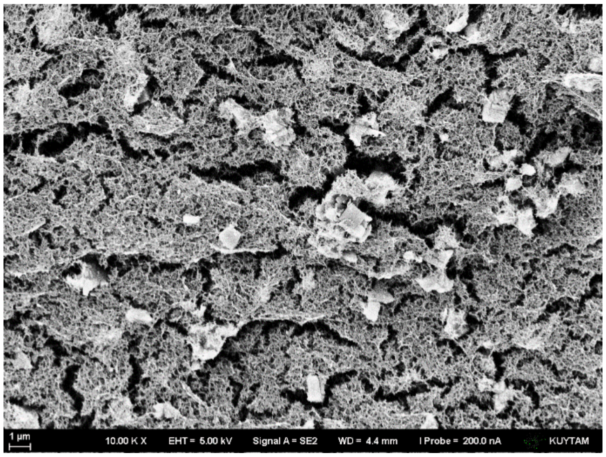

(d) AlgMIL160-3-3 (EHT=5kV, 10.00 KX)

Figure S5. SEM images for AlgMIL160-3-1 and AlgMIL160-3-3.

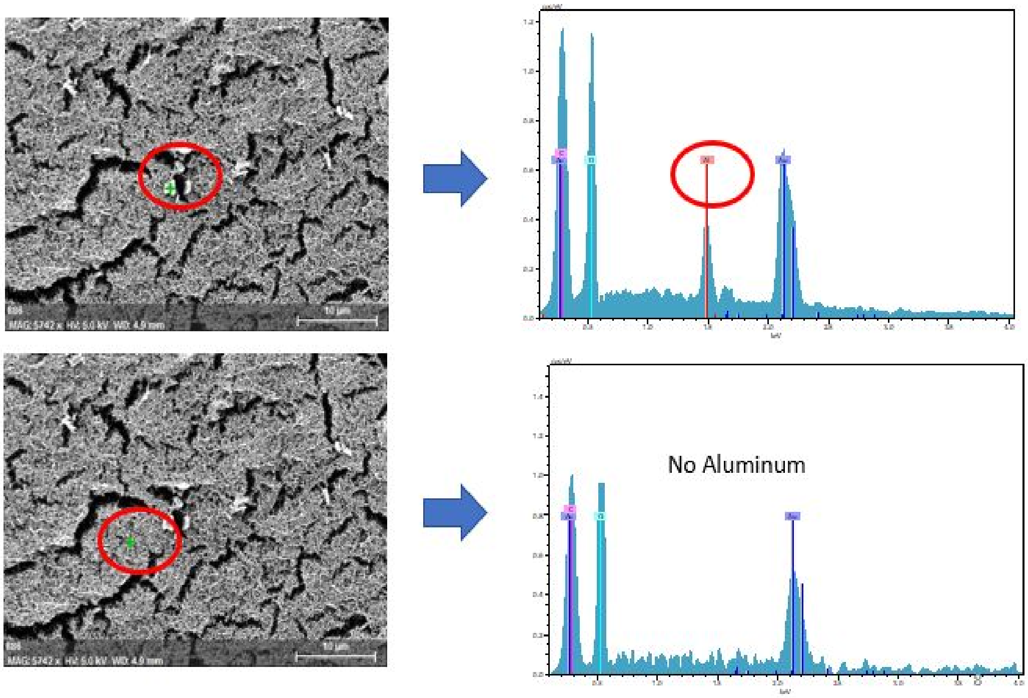

Figure S6. SEM/EDX of AlgMIL160-3-1 showing the presence of aluminum in MOF crystalline particles.

4. CO_2 uptake calculations for adsorption column

Mole balance for CO_2 in the gas phase was performed to determine the total CO_2 uptake on the MOFACs. The total CO_2 uptake by the column can be calculated using equation (1) as given in the following:

$$Q = \left(\frac{P}{RT}\right)\left[\int_0^{t_\infty} v_{N_2,in}\left(\left(\frac{y^E_{CO_2,out}(t)}{1-y^E_{CO_2,out}(t)}\right) - \left(\frac{y_{CO_2,out}(t)}{1-y_{CO_2,out}(t)}\right)\right)dt\right] \quad (1)$$

Where Q is the total adsorbed CO_2. P , R, T are pressure inside the column, ideal gas constant, and temperature of the bed, respectively. $v_{N_2,in}$ is the volumetric flow rate of the inlet nitrogen. $y^E_{CO_2,out}(t)$ and $y_{CO_2,out}(t)$ are the mole fraction of CO_2 in the outlet when the bed is run at empty and filled mode, respectively.

5. Langmuir isotherm parameters and IAST selectivity

Table S3. Single site Langmuir isotherm model parameters for CO_2 and N_2 adsorption on MOFACs with different MOF content at 25 ℃ for gas mixture of 15% CO_2/85% N_2.

* In this work, single site Langmuir model was used to fit the experimental isotherm data for pure CO_2 and N_2 at 25 ℃. $q_i = q_m \frac{K_l P_i}{1+K_l P_i}$

Sample	MOF content wt.%	Component	q_m (mmol/g)	K_l (mbar-1)	R^2	IAST Selectivity (CO_2/N_2)*
AlgMIL160-3-1	25	CO_2	3.07	1.11E-03	1.00	61.4
AlgMIL160-3-1	25	N_2	1.17	5.46E-05	0.99	
AlgMIL160-3-3	50	CO_2	3.87	1.48E-03	1.00	70.7
AlgMIL160-3-3	50	N_2	1.43	6.90E-05	0.99	
AlgMIL160-3-9	75	CO_2	5.34	1.53E-03	1.00	69.7
AlgMIL160-3-9	75	N_2	2.05	6.92E-05	0.99	
MIL-160(Al)	100	CO_2	6.65	1.56E-03	0.99	52.9
MIL-160(Al)	100	N_2	2.12	1.20E-04	0.99	
Ca-alginate	0	CO_2	0.76	6.47E-04	1.00	13.8
Ca-alginate	0	N_2	0.76	4.66E-05	0.99	

The selectivity was calculated using IAST at 25 ℃ and 1000 mbar for 15% CO_2/85% N_2 gas mixture.

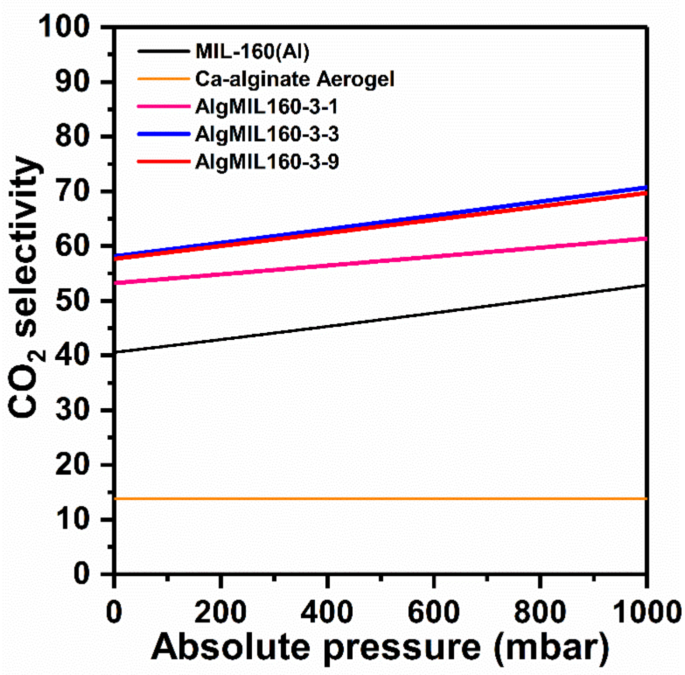

Figure S7. CO_2/N_2 IAST selectivity at 25 °C for the gas mixture of 15% CO_2/85% N_2.

6. Breakthrough experiments over composites from 15% CO_2/85% N_2 at 25 and 50 ˚C

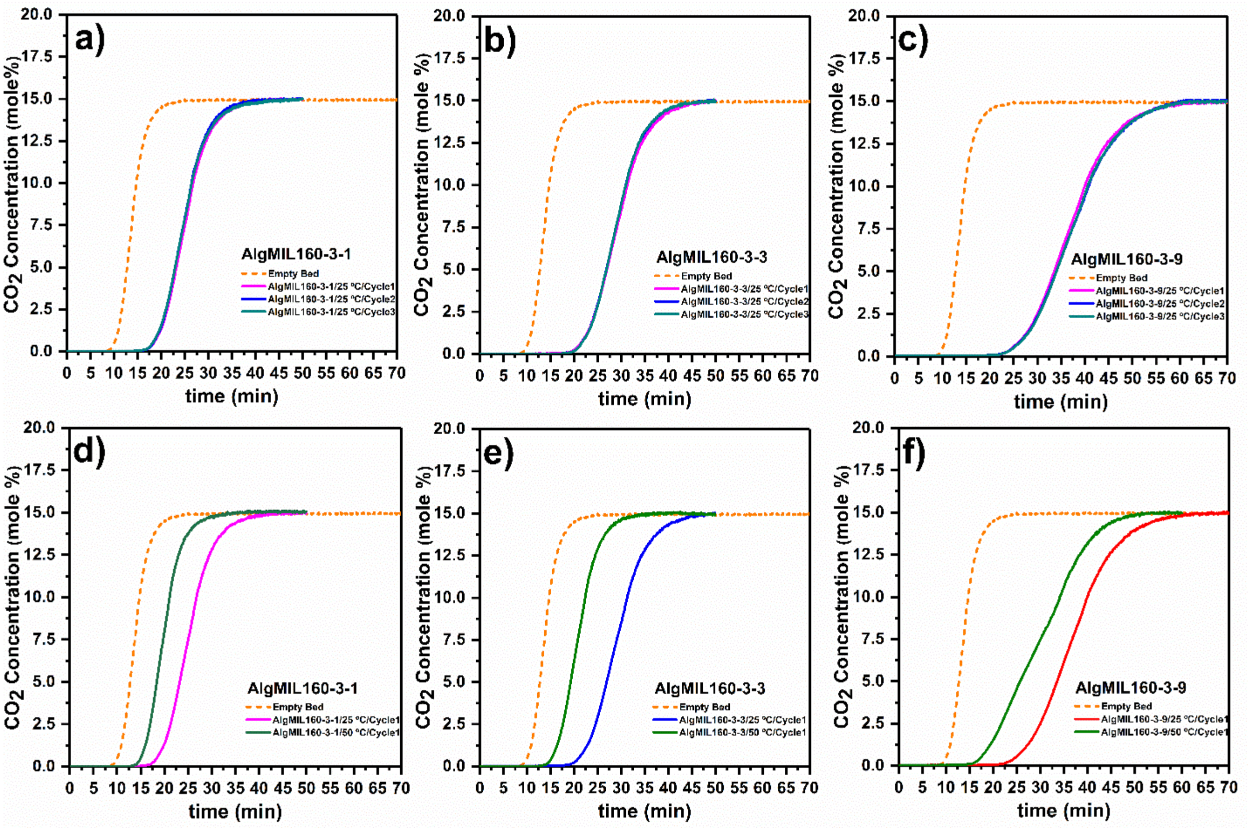

Figure S8. Reproducibility of dynamic adsorption experiments with 3 repeated runs for each composite: (a) AlgMIL160-3-1, (b) AlgMIL160-3-3, and (c) AlgMIL160-3-9. The breakthrough curves obtained at 25 ˚C and 50 ˚C for each composite: (d) AlgMIL160-3-1, (e) AlgMIL160-3-3, and (f) AlgMIL160-3-9.

Table S4. Equilibrium CO_2 uptake of MOFACs at 25 ˚C from pure CO_2 at 150 mbar (for single component static mode) and binary mixture of 15% CO_2/85% N_2 (for binary dynamic adsorption mode) at 1000 mbar.

Sample	Temperature	MOF Loading	Adsorbent amount (after test)	CO_2 Uptake			
				Binary dynamic adsorption (Exp.)	Single component adsorption (Static Exp.)	Single site Langmuir model	Binary IAST model
	˚C	wt.%	g	mmol/g	mmol/g	mmol/g	mmol/g
AlgMIL160-3-1	25	0.25	0.81	0.44	0.43	0.44	0.43
AlgMIL160-3-3	25	0.5	0.89	0.55	0.70	0.71	0.68
AlgMIL160-3-9	25	0.75	0.86	0.86	0.98	1.00	0.97

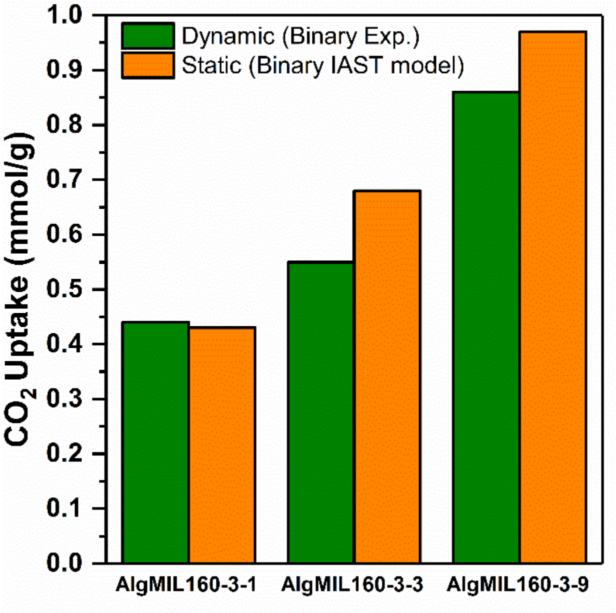

Figure S9. Comparison of CO_2 uptake of MOFACs between experimental dynamic mode and Binary IAST model of static mode.

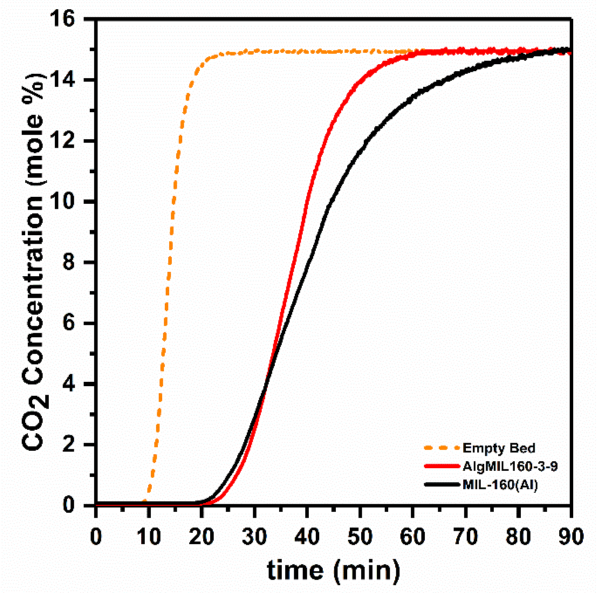

Figure S10. Comparison of CO_2 breakthrough of pure MIL-160(Al) in powder form and AlgMIL160-3-9 at 25 °C.
